# Silver vacancy concentration engineering leading to the ultralow lattice thermal conductivity and improved thermoelectric performance of Ag_1-x_InTe_2_

**DOI:** 10.1038/s41598-019-55458-3

**Published:** 2019-12-11

**Authors:** Yaqiong Zhong, Yong Luo, Xie Li, Jiaolin Cui

**Affiliations:** 1 School of Materials Science and Engineering, China University of Mining and Technology, Xuzhou, 221116 China; 2 School of Materials and Chemical Engineering, Ningbo University of Technology, Ningbo, 315211 China

**Keywords:** Thermoelectrics, Applied physics

## Abstract

AgInTe_2_ compound has not received enough recognition in thermoelectrics, possibly due to the fact that the presence of Te vacancy (V_Te_) and antisite defect of In at Ag site (In_Ag_) degrades its electrical conductivity. In this work, we prepared the Ag_1-*x*_InTe_2_ compounds with substoichiometric amounts of Ag and observed an ultralow lattice thermal conductivity (*κ*_L_ = 0.1 Wm^−1^K^−1^) for the sample at *x* = 0.15 and 814 K. This leads to more than 2-fold enhancement in the ZT value (ZT = 0.62) compared to the pristine AgInTe_2_. In addition, we have traced the origin of the untralow *κ*_L_ using the Callaway model. The results attained in this work suggest that the engineering of the silver vacancy (V_Ag_) concentration is still an effective way to manipulate the thermoelectric performance of AgInTe_2_, realized by the increased point defects and modified crystal structure distortion as the V_Ag_ concentration increases.

## Introduction

Thermoelectrics (TE) materials can transfer energy from heat to electricity or vice versa due to their inherent Seebeck or Peltier effects. However, the TE device at present is still inefficient up to date mainly due to low TE performance or ZT valueof the materials, which is defined as ZT = *α2σT*/κ,where *α*, *σ*, *T*, and *κ* are each the Seebeck coefficient, electrical conductivity, absolute temperature, and total thermal conductivity consisting of electronic (*κ*_e_) and lattice (*κ*_L_) components. Therefore, it is quite necessary to explore novel TE candidates. Ternary I-III-VI_2_ (I = Cu, Ag; III = Al, Ga, In; VI = S, Se, Te) chalcogenides, such as CuGaTe_2_^1^, CuInTe_2_^2^, AgInSe_2_^3^ and AgGaTe_2_^4,5^, have recently attracted much attention in thermoelectrics because of their unique crystal and band structures^6,7^, among which the cation vacancy and crystal structure are two important factors in engineering both the carrier concentration and phonon transport^8–10^. However, to our best knowledge, AgInTe_2_ has not received due recognition. The possible reason is the presence of the defects V_Te_ and In_Ag_ in AgInTe_2_, which were identified through analyzing the electrical data of AgInTe_2_ by Bellabarba etc^11^. These two defects, which are vital to the formation of two shallow donor levels at around 9 mV and 28 mV below the conduction band minimum (CBM)^11^, directly degrade the electrical conductivity (10^3^~10^6^ mΩcm at 300–700 K)^12,13^. As a consequence, the highest ZT value of AgInTe_2_ is only 0.07 at 600 K^13^. The temperature at which the highest ZT value was attained is just about half the melting point of AgInTe_2_ (see the phase diagram in Fig. S1). In this regard, it is important to eliminate the defects V_Te_ and/or In_Ag_ in order to improve the TE performance of AgInTe_2_.

On the other hand, the Debye temperature (*Θ*_D_) and average sound velocity of AgInTe_2_ are 113–202 K and *ν*_age_ = 1240 ms^−1^ respectively^11,14,15^, which are very low compared to those of CuInTe_2_ (*Θ*_D_ = 197.5 K; *ν*_age_ = 3420 ms^−1^)^16^ and CuGaTe_2_ (*Θ*_D_ = 229.0 K; *ν*_s_ = 2072.0 ms^−1^, *ν*_l_ = 3817.0 ms^−1^)^1^. Besides that, the average sound velocity is only about half that of AgGaTe_2_ (*ν*_s_ = 1812.0 ms^−1^, *ν*_l_ = 2990.0 ms^−1^)^7^. It implies that the compound AgInTe_2_, naturally, has a low lattice thermal conductivity^17^, hence it is potentially a good TE candidate. Apart from that, AgInTe_2_ has a relatively less silver vacancy formation enthalpy (∆*H*_V_(Ag) = 2.90 eV) than most ternary I-III-VI_2_ chalcogenides, except for CuInTe_2_ (∆*H*_V_(Cu) = 2.59 eV) and CuGaTe_2_ (∆*H*_V_(Cu) = 2.73 eV)^18^. Therefore, from the thermodynamics point of view, AgInTe_2_ with substoichiometric amounts of Ag should be more stable than most of them. The presence of substoichiometric Ag eliminates the V_Te_ and forms the silver vacancy V_Ag_ and thereby might increase the point defect scattering of phonons while improving the electrical conductivity simultaneously^5^.

Motivated by the above priorities of AgInTe_2_ with substoichiometric amounts of Ag in thermoelectrics, we believe that there is still some room to improve its TE performance. Therefore, in this work we prepared Ag_1-*x*_InTe_2_ (here the *x* value representing the Ag deficiency, and the Ag vacancy concentration (V_Ag_), V_Ag_ = *x*/2) and then examined their TE performance in the range from room temperature (~RT) to ~820 K.

## Experimental Procedures

### Syntheses and preparation of samples

The three elements Ag, In and Te, were loaded into different vacuum silica tubes, according to the chemical compositions of Ag_1-*x*_InTe_2_ (*x* = 0, 0.05, 0.1, 0.15 and 0.2). They were then heated to 1073 K in 30 min and held at this temperature for 48 h. After that, the ingots were cooled down to 873 K in 8 h before being quenched in water. Subsequently, they were annealed at 553 K for 72 h. After cooling to RT, the ingots were ball milled for 5 h, and the dried powders were then rapidly sintered using the spark plasma sintering apparatus (SPS-1030) specifically programmed with a peak temperature of 823 K and pressure of 55 MPa. The densities of the sintered bulks were measured by using the Archimedes’ method. The bulk samples about the size of 2.5 × 3 × 12 mm^3^ were prepared for electrical property measurements, and those of ϕ10 × 1.5 mm^2^ for thermal diffusivity measurement.

### Analyses and measurements

The Hall coefficient (*R*_H_) at RT were measured by using a PPMS (Model-9) with a magnetic field sweeping between ± 5.0 T. The Hall mobility (*μ*) and carrier concentration (*n*_H_) were subsequently determined based on the equations *μ* = |*R*_H_|*σ* and *n*_H_ = 1/(e*R*_H_), respectively (*e* is the electron charge).

The Seebeck coefficients and electrical conductivities were evaluated by using ZEM-3 under a helium atmosphere at a temperature ranging from ~RT to ~820 K, with an uncertainty of <5.0% for each. The thermal diffusivities were measured by using TC-1200RH, with an uncertainty of <10.0%. The heat capacities (*C*_p_) of the present materials were estimated by using the Dulong–Petit rule, C_p_ = 3*n*R (here *n* is the number of atoms per formula unit and *R* is the gas constant). The C_p_ estimation is used in AgInTe_2_ or its related systems by many works^13,19,20^, and is proved to be in a good agreement with (or only ~6% higher than) the measured one^13,20^, as the C_p_ values above Debye temperature (*Θ*_D_) are almost unchanged^21^. When calculating the electronic thermal conductivities (*κ*_e_) according to the equation *κ*_e_ = *LσT*, the Lorenz numbers *L* were estimated by using the formula *L* = 1.5 + exp(−|*α*|/116)^22^, (where *L* is in 10^−8^ W Ω K^−2^ and *α* in *μ*V K^−1^). The total uncertainty for ZT was ~20%.

### Analyses

The chemical compositions were determined by using an electron probe micro-analyzer (EPMA) (S-4800, Hitachi, Japan) with an accuracy of >97%.

The powder X-ray diffraction patterns of the samples were registered by using X-ray powder diffractometer (XRD) (D8 Advance) operating at 50 kV and 40 mA in a step size of 0.02° in the range of 10° to 100°, and a X’Pert Pro, PANalytical code was used to do the Rietveld refinement of the XRD patterns with a step size of 0.01° using the same operating voltage and current. The lattice constants *a* and *c* were directly obtained from the refinement of the X-ray data using the Jade software.

Differential scanning calorimeter (DSC) is conducted in a Netzch STA 449 F3 Jupiter equipped with a TASC414/4 controller. The instrument is calibrated from a standard list. The sample of the powder (*x* = 0.15) is loaded into an open alumina crucible. The measurement is performed between ~300 K to ~820 K with a heating rate of 5 K min^−1^ in Ar atmosphere.

## Results and Discussion

### Chemical compositions and X-ray diffraction patterns

The scanning electron microscopy (SEM) images of both polished and freshly fractured surface, three mappings of elements (Ag, In, Te) as well as the energy-dispersive x-ray spectroscopy (EDS) of the sample at *x* = 0.15 are shown in Fig. S2a–f. The analyzed chemical compositions of substoichiometric Ag_1-*x*_InTe_2_ (*x* = 0.15) are shown in Table S1, where the number of Te moles is normalized to 2.0. From the analyzed results, it is observed that there is a slight Te evaporation during the sample preparation process. Further, the element distribution is not uniform in the matrix, mainly due to the precipitation of the second phases. Also, the densities of the sintered bulks Ag_1-*x*_InTe_2_ (*x* = 0~0.2), ranging from 5.92 × 10^3^ kg/m^3^ (*x* = 0) to 5.97 × 10^3^ kg/m^3^ (*x* = 0.2), are about ~96.0% of the theoretical one (6.17 × 10^3^ kg/m^3^) of AgInTe_2_. The low density of the materials is detrimental to the electrical conductivity, even though it is helpful to scatter phonons.

The refinements of the powder X-ray diffraction (XRD) patterns of the four Ag_1-*x*_InTe_2_ samples (*x* = 0, 0.1, 0.15, 0.2) are shown in Fig. S3. The results involving the crystallographic data, Wyckoff positions, atomic coordinates and site occupancy factors (SOFs) are listed in Table S2-5, where the SOFs of Ag (4a), In (4b) and Te (8d) are all 1.0 in AgInTe_2_ without the antisite defect In_Ag_ identified. In addition, the main peaks of the patterns are indexed to the existing chalcopyrite AgInTe_2_ (PDF: 65–0355) with the minor impurities AgTe_3_ (1.3 wt.% at *x* = 0) and Te (less than 3.1 wt.% at *x* = 0, 0.15, 0.2) precipitated. In order to prevent the precipitation of the impurities AgTe_3_ and Te from the main phase AgInTe_2_, all the samples were subject to heat treatment at 813 K for 72 h. After the heat treatment, the materials were purified without any impurity phases in the samples at *x* = 0–0.1, and only trace Te element still exists in the samples at *x* = 0.15 and 0.2, as shown in the refined XRD patterns in Fig. S4. The crystallographic data, Wyckoff positions, atomic coordinates and site occupancy factors (SOFs) in the heat-treated samples are not shown here.

**Figure 1:**
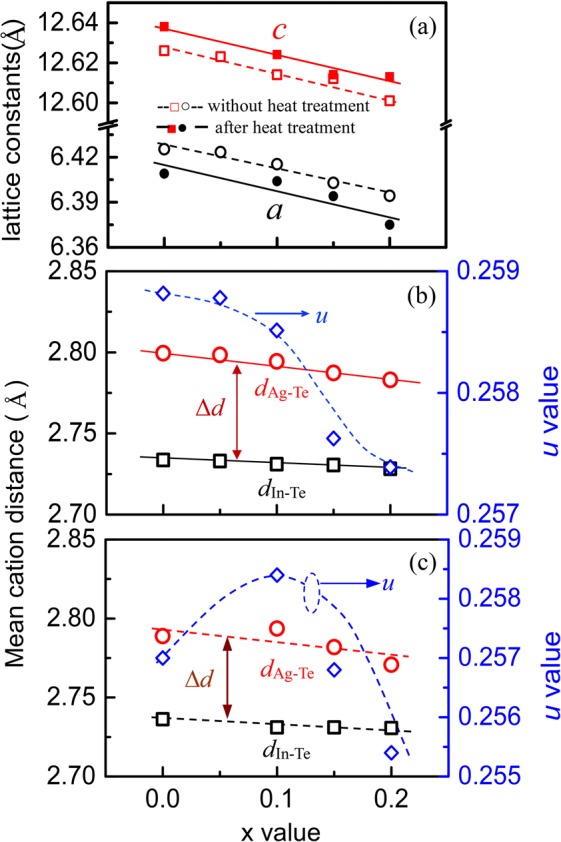
(**a**) Lattice constants *a* and *c* as a function of *x* value in Ag_1-*x*_InTe_2_ for the samples with or without heat treatment. (**b**,**c**) Structural parameters involving cation (anion)-Te distances (*d*_Ag-Te_, *d*_In-Te_) and anion position displacement parameter (*u*) as a function of *x* value in Ag_1-*x*_InTe_2_. (**b**) For the samples without heat treatment; (**c**) For the samples with heat treated at 813 K for 72 h.

Figure 1a presents the lattice constants as a function of *x* value for the Ag_1-*x*_InTe_2_ samples with or without heat treatment, where it is observed that the lattice constants *a* and *c* of two set samples decrease almost linearly, following the Vegard’s law. This is indicative of the shrinkage of the crystal structure. Apart from that, the tetragonal distortion parameter *η* value (*η* = *c*/2*a*) of non-heat treated samples increases from 0.9825 (*x* = 0) to 0.9853 (*x* = 0.2) (see Tables S2, 6) and the anion position displacement parameter *u* decreases from 0.2588 (*x* = 0) to 0.2573 (*x* = 0.2) as the *x* value increases. The *u* value of AgInTe_2_ is less than the reported value of 0.2618^23^. Besides, the difference (∆*d*) between the cation and anion distances (∆*d* = *d*_Ag-Te_ − *d*_In-Te_) tends to be small (see Fig. 1b). A similar tendency was also observed in the heat-treated samples, as shown in Fig. 1c and Table S6, where the *u* value starts to decrease above *x* = 0.1. These results suggest that the crystal structure distortion of Ag_1-*x*_InTe_2_ exists (*u* > 0.25) but tends to be weakened as the cation Ag vacancy concentration increases, in spite of the presence of a minor impurity Te.

### Transport properties and TE performance

**Figure 2:**
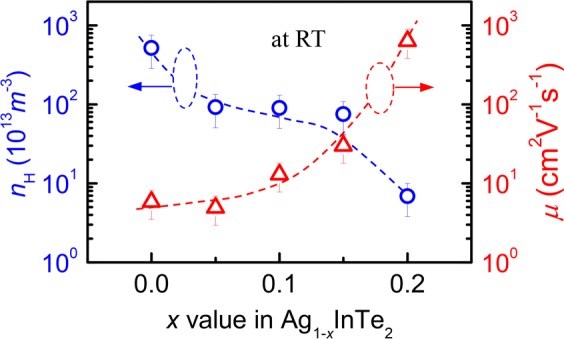
Hall carrier concentration (*n*_H_) and mobility (*μ*) at RT as a function of *x* value in Ag_1-*x*_InTe_2_ without heat treatment.

In order to gain a better understanding of the effect of the crystal structure and Ag vacancy engineering on the transport properties, we have measured the Hall coefficients (*R*_H_) at RT for non-heat treated samples, and then calculated the Hall carrier concentrations (*n*_H_) and mobility (*μ*). The results are shown in Fig. 2, in which the *n*_H_ value generally decreases from 5.19 × 10^16^ cm^−3^ to 6.9 × 10^14^ cm^−3^ as the *x* value (or V_Ag_) increases, and that the *μ* value increases from 5.83 cm^2^/Vs to 636.7 cm^2^/Vs. This indicates that Ag cation vacancy has a negative effect on the carrier concentration. Such an effect, which has been reported in many materials, such as Cu-Ga(In)-Te ternary compounds^24–26^, can be elucidated by unpinning the Fermi level (*E*_f_) followed by its movement towards the conduction band (CB)^27,28^. As a consequence, the p-type holes is neutralized and the carrier concentration is thus reduced. Although the *R*_H_ values might be affected by the presence of the Te and/or AgTe_3_, it is believed that the impact should be minimum, due to the limited contents of the impurities. Plus, the *n*_H_ value is rather low compared to the reported counterparts CuInTe_2_ (>1.87 × 10^18^ cm^−3^)^29^ and CuGaTe_2_ (1.1 × 10^18^ cm^−3^)^1^ but is comparable to that of AgGaTe_2_ (1.13 × 10^16^ cm^−3^)^5^. However, the mobility *μ* value (87.0 cm^2^/Vs) of AgGaTe_2_ at RT is much higher than that of AgInTe_2_, which implies that AgInTe_2_ has a much larger electrical resistivity than AgGaTe_2_ (~1.0 × 10^2^ mΩ cm at RT)^5^, even though the bandgap of AgInTe_2_ (*E*_g_ = 1.04 eV)^12^ is less than that of AgGaTe_2_ (*E*_g_ = ~1.2 eV)^30^.

**Figure 3:**
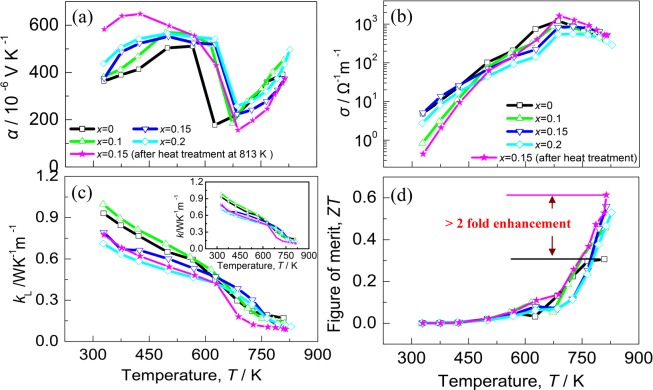
Thermoelectric performance of Ag_1-*x*_InTe_2_ without heat treatment as a function of temperature for different *x* values. (**a**) Seebeck coeffcients (*α*) with different *x* values; (**b**) Electrical conductivities (*σ*) with different *x* values; (**c**) Lattice thermal conductivities (*κ*_L_) with different *x* values, an inset is the total thermal conductivities (*κ*); (**d**) ZT values as a function of temperature. Besides that, the TE performance of the sample at *x* = 0.15 after heat treatment at 813 K for 72 h was presented for comparison.

The thermoelectric performance of Ag_1-*x*_InTe_2_ before heat treatment as a function of temperature are presented in Fig. 3(a–d), where the Fig. 3a presents the temperature dependence of the Seebeck coefficients (*α*). All the *α* values are positive, indicating that the materials exhibit a p-type semiconductor behavior. At first, the *α* values for most samples increase as the temperature increases up to ~620 K, and then they rapidly decrease until at 660 K. Above ~660 K the *α* values increase again as the temperature increases. Such a zigzag-shaped relation is likely attributed to the phase transition in the materials, which will be discussed below.

Figure 3b presents the electrical conductivities (*σ*) as a function of temperature, where the *σ* values increase with the increase of the temperature, but they slightly decrease as the *x* value increases. At 725 K, the *σ* value of the sample at *x* = 0.15 reaches the highest (8.56 × 10^2^ Ω^−1^m^−1^), and then it decreases to 5.15 × 10^2^ Ω^−1^m^−1^ at 814 K.

The lattice thermal conductivities (*κ*_L_) are displayed in Fig. 3c. Generally, they reduce as the temperature increases. More specifically, the *κ*_L_ values reduce rapidly when the temperature is higher than ~620 K; however, above 660 K they reduce relatively slowly. The abrupt changes in *κ*_L_ are similar to those observed in the Seebeck coefficients, confirming the phase transitions at these two temperatures. Further, the lattice part (*κ*_L_) at ~814 K is 0.1 WK^−1^m^−1^ for the sample at *x* = 0.15, about a half that at *x* = 0. Generally, the *κ*_L_ values are rather low compared to the reported counterparts CuInTe_2_^2,9,10,31,32^ and CuGaTe_2_^33–35^ but are comparable to those in AgGaTe_2_^4^. The total *κ* values of the samples, which are presented in Fig. 3c as an inset, bear resemblance to the lattice parts (*κ*_L_), suggesting that the phonon transport dominates the heat conduction.

Combined with the above three parameters (*α*, *σ*, *κ*), we obtain the ZT values shown in Fig. 3d. The ZT values, which are less than 0.2 for all the samples below 700 K, rapidly increase with the increase of the temperature. At 814 K the ZT value reaches 0.55 for the sample at *x* = 0.15. This value is about 1.8 times that of the pristine AgInTe_2_ with no heat treated.

One of the factors that contributes to the enhancement in the ZT value in this work is the higher measuring temperature (~814 K) than the reported one (~600 K)^11,13^, above which the TE performance of the materials are seldom reported in the previous documents, likely due to the thermal instability issue.

**Figure 4:**
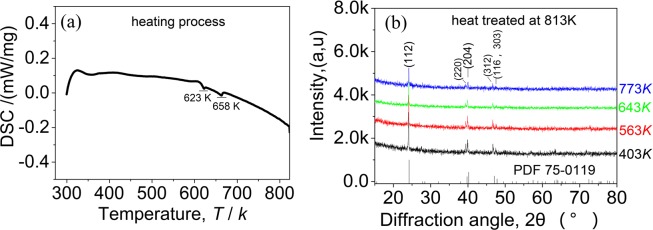
(**a**) Differential scanning calorimetry (DSC) signal as a function of temperature for the material Ag_0.85_InTe_2_ with heat treatment, where two exothermic effects at 623 K and 658 K were observed in the heat processing; (**b**) High temperature diffraction XRD patterns of heat treated material Ag_0.85_InTe_2_.

In order to gain a deep understanding of the thermal stability of the materials, the DSC analysis of the material Ag_1-*x*_InTe_2_ (*x* = 0.15) was made in the heating process. The result is shown in Fig. 4a, in which we have observed two endothermic effects at ~623 K and 658 K, corresponding to the phase transitions mentioned above. Although the high temperature XRD patterns (403K–773K), shown in Fig. 4b, are insufficient to demonstrate the phase transitions, as all the diffraction peaks can be indexed to the chalcopyrite AgInTe_2_ (PDF:75-0119) with no visible movement of the peak positions, we can not rule out the possibility that the materials have undergone order-disorder transitions. These transitions are formed due to the Ag-In antisite occupancy starting in the critical temperatures^11^, as was observed in the Cu-containing I-III-VI_2_ ternary chalcopyrites^36–38^. At around ~620 K, the partial chalcopyrite structure (assumed to be the α phase) is transited to sphalerite structure (β phase) followed by the co-existence of the two phase field (α + β) in the temperature range from 620 K to 655 K. The transition from the α to β phase likely results from the formation of In_Ag_ antisite defect. However, above ~655 K, the high energy allows all the α phase to be transformed to the β phase. Alternatively, the β phase returns to the α phase above 655 K due to the dissolvation of the antisite defect In_Ag_. The order-disorder transformation was also observed in the ZnTe-doped CuInTe_2_ system^39^. However, it is very difficult to unravel its nature at the moment, and a detailed investigation and analysis is therefore necessary. Anyhow, what is certain is that the abrupt changes of the Seebeck coefficients at around 620 K and 660 K should be caused by the phase transitions, rather than by the precipitation of the impurity phase Te and/or AgTe_3_ since all the samples have similar transitions at these temperatures, including the sample at *x* = 0.1 with no impurity phases precipitated (see Fig. S3b). Although the heat-treated sample at *x* = 0.15 was purified with only trace impurity Te in the matrix, it exhibits the similar temperature dependence of the TE performance to the non-treated samples (see Fig. 3a–d). In addition, the heat-treated sample at *x* = 0.15 gives the highest ZT value of 0.62 at 814 K, more than 2 times that of the pristine AgInTe_2_.

Another contribution to the remarkable improvement in TE performance at high temperatures results from the extremely low lattice part (*κ*_L_ = 0.1 WK^−1^m^−1^ for the sample *x* = 0.15), as the highest power factors for the samples, which range from 7.2 μW/cm-K^2^ to 8.3 μW/cm-K^2^ around 814 K, are almost the same (see Fig. S5). The low lattice part (*κ*_L_) is in a good agreement with the glass thermal conductivity ~0.11 WK^−1^m^−1^ estimated by using the Cahill formula^1,40^ below:

1 $${\kappa }_{{\rm{\min }}}\sim 1.2\frac{{k}_{B}}{{\Omega }^{2/3}}\frac{{v}_{l}+2{v}_{{\rm{s}}}}{3}$$

Here, the *k*_B_, *Ω*, *v*_l_ and *v*_s_ are the the Boltzmann constant, volume per atom, longitudinal and shear sound velocities. In this estimation, however, the average of the speed of sound (*ν*_m_) is used. Therefore, the *κ*_min_ of AgInTe_2_ (~0.11 WK^−1^m^−1^) can be easily deduced from the *κ*_min_ value (0.18~0.23 WK^−1^m^−1^) of AgGaTe_2_^7^, by using the *ν*_m_ (1240 m/s) and Debye temperature *Θ*_D_ (156 K) of AgInTe_2_ and *ν*_m_ (2402 m/s) and *Θ*_D_ (192 K) of AgGaTe_2_^7^. The ultralow lattice part in Ag_1-*x*_InTe_2_ is largely attributed to the enhanced phonon scattering on the point defects due to the increased concentration of the silver vacancy (V_Ag_).

In order to confirm the dominant effect of the silver vacancy on the unexpected low *κ*_L_, we performed a theoretical calculation of the lattice thermal conductivity based on the Callaway model^41^, under the assumption that the Umklapp and point defect scatterings are the main scattering mechanisms. In this case, the ratio (*κ*_L,F_/*κ*_L0_) of the modeled lattice thermal conductivity of the V_Ag_-containing samples, *κ*_L,F_, to that of the experimentally determined lattice thermal conductivity without V_Ag_, *κ*_L0_, is shown in Eq. 2,

2 $$\frac{{\kappa }_{L,F}}{{\kappa }_{L0}}=\frac{{\tan }^{-1}(u)}{u}\,\,{u}^{2}=\frac{{\pi }^{2}\Theta {}_{D}\Omega }{\hslash {{V}_{m}}^{2}}{\kappa }_{L0}\Gamma $$

Here *u* and *Г* are the disorder scaling parameter and the disorder scattering parameter. The ν_m_, and *ћ* are the mean sound velocity and Planck constant. We use the factor *Г* below (Eq. 3)^9^ to predict the *κ*_*L*,*F*_ values for the AgInTe_2_-based chalcogenides, and the related parameters are presented in Table 1.

3 $$\Gamma ={x}_{i}(1-{x}_{i})[{(\frac{\Delta {{M}}_{{i}}}{M})}^{2}+\varepsilon {(\frac{\Delta {\delta }_{{i}}}{{x}_{i}\delta })}^{2}]$$

**Table 1:** Parameters used for estimating the lattice thermal conductivity (*κ*_L_) at *x* = 0.15 using Callaway model.

Symbol	Representation
Θ_D_	Debye temperature, 156 K (ref. ^13^)
ν_m_	Average sound velocity, 1240 ms^−1^ (ref. ^13^)
ћ	Planck constant
Ω	Average volume per atom
*Ɛ*_0_	9.2 (ref. ^13^)
*Ɛ*	15.64 (ref. ^13^)

In Eq. 3, the parameters *x*_i_, ∆*M*_*i*_*/M* and Δ*δ*_i_/*δ* are the molar fraction of Ag, mass difference, and the local change in lattice parameters.

**Figure 5:**
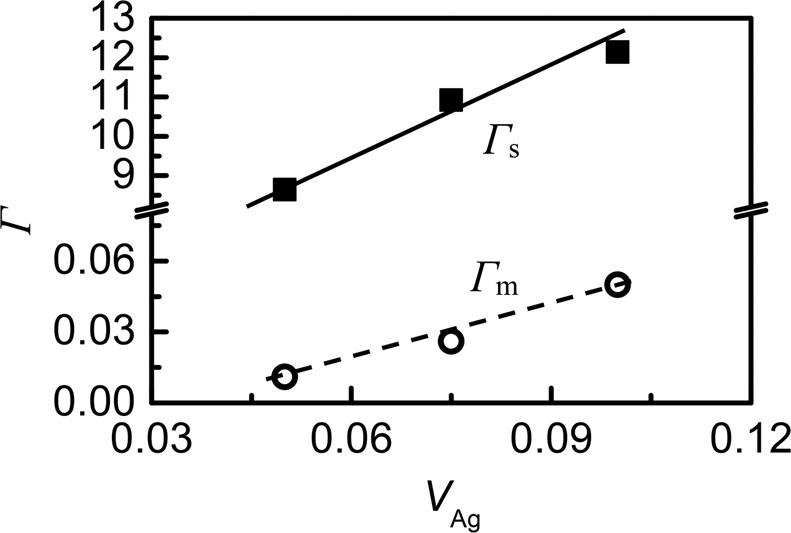
The mass fluctuations *Γ*_m_ and strain field fluctuations *Γ*_s_ for three samples at *x* = 0.1, 0.15 and 0.2 as a function of silver vacancy V_Ag_ (V_Ag_ = *x*_i_/2).

Based on the above calculations, the mass fluctuations *Γ*_m_ and strain field fluctuations *Γ*_s_ for three samples at *x* = 0.1, 0.15 and 0.2 are displayed in Fig. 5 as a function of silver vacancy (V_Ag_ = *x*_i_/2), while the fitted *κ*_*L*,*F*_ values, the disorder scaling parameter (*u*) and the disorder scattering parameter (*Γ*) consisting of *Γ*_m_ and *Γ*_s_ are presented in Table 2. It is observed that the strain field fluctuations *Γ*_s_ is mainly responsible for the lattice disorder, while the mass fluctuations *Γ*_m_ plays a minor role. The fitted *κ*_L,F_ values are roughly in accordance with the experimentally determined *κ*_L_ values, except for those at high temperatures, which are about 30–60% higher than the measured ones at 814 K (see Table 2). The origin for the high estimated *κ*_L,F_ values is due to the fact that the extra phonon scattering on the distorted crystal structure is not taken into account. This distortion, which is caused by the different interactions between the bonds Ag-Te and In-Te and represented by the higher anion position parameter *u* (*u* ≈ 0.258 > 0.25, in Fig. 1b,c), has a profound impact on the lattice part *κ*_L_^42,43^. However, the phonon scattering on the increased phase boundaries due to the presence of the impurity phases (AgTe_3_ and Te) should play a minor role, as it acts only at low to middle temperatures.

**Table 2:** Calculated lattice thermal conductivities (κ_L.cal_.) of the sample at x = 0–0.2 from the experimental κ_L.exp_. at x = 0.

Tem. (K)	*x* = 0 V_Ag_ = 0	*x* = 0.1, V_Ag_ = 0.05		*x* = 0.15, V_Ag_ = 0.075		*x* = 0.2, V_Ag_ = 0.1	
		*Г* = 8.67, *U* = 83.91		*Г* = 10.94, *U* = 94.26		*Г* = 12.15, *U* = 99.35	
	*κ*_L.exp._	*κ*_L.exp._	*κ*_LF._	*κ*_L.exp._	*κ*_LF_	*κ*_L.exp._	*κ*_LF_
328	0.93	0.99	0.99	0.79	0.88	0.71	0.84
374	0.84	0.89	0.89	0.66	0.80	0.64	0.76
499	0.65	0.71	0.69	0.60	0.62	0.51	0.59
567	0.59	0.61	0.63	0.53	0.56	0.46	0.53
626	0.45	0.51	0.48	0.47	0.43	0.42	0.41
725	0.22	0.22	0.23	0.31	0.31	0.27	0.27
764	0.19	0.16	0.20	0.17	0.18	0.17	0.17
814	0.17	0.14	0.18	0.10	0.16	0.12	0.16

Having figured out the phonon scattering mechanism, we thus propose that the low lattice part *κ*_L_ at high temperatures mainly results from the Umklapp scattering, extra point defect and crystal structure distortion. However, the phonon scattering by the carriers weakens as the carrier concentration (*n*_H_) reduces, which neutralizes the increased phonon scattering to some extent.

## Conclusions

In this work we prepared the Ag_1-*x*_InTe_2_ (*x* = 0–0.2) with substoichiometric amounts of Ag and examined their thermoelectric performance. The analyses reveal that the main phase AgInTe_2_ undergoes phase transitions at ~620 K and ~655 K, even though there are minor impurity phases (element Te and/or AgTe_3_) precipitated. Upon the increasing of silver vacancy (V_Ag_) concentration, an ultralow lattice thermal conductivity (*κ*_L_ = 0.1 Wm^−1^K^−1^) at *x* = 0.15 was attained, and a more than 2-fold enhancement in ZT value achieved at 814 K compared to the pristine AgInTe_2_. Besides, the origin of the ultralow lattice part *κ*_L_ is decoupled and elucidated using the Callaway model, proving that the silver vacancy concentration engineering is still an effective way to manipulate the thermoelectric performance of AgInTe_2_.

## Supplementary Information

Supplementary information

## Data Availability

The datasets generated during and/or analyzed during the current study are available from the corresponding author on reasonable request.
