# Supplementary material for: Silver vacancy concentration engineering leading to the ultralow lattice thermal conductivity and improved thermoelectric performance of Ag1-xInTe2
Source: Sci Rep. 2019 Dec 11;9:18879. doi: 10.1038/s41598-019-55458-3 (PMC6906449; doi:10.1038/s41598-019-55458-3)
Supplement: Supplementary file 1 — Supplementary information [file 41598_2019_55458_MOESM1_ESM.pdf]

Supporting Information

Silver vacancy concentration engineering leading to the ultralow lattice thermal conductivity and improved thermoelectric performance of $Ag_{1-x}InTe_2$

Yaqiong Zhong,[a,b] Yong Luo,[a] Xie Li,[b] Jiaolin Cui[b*]*

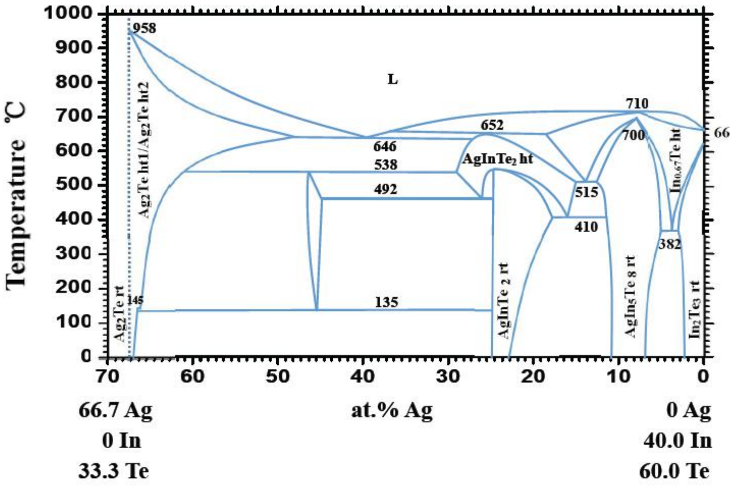

Figure S1 Phase diagram of Ag-In-Te.

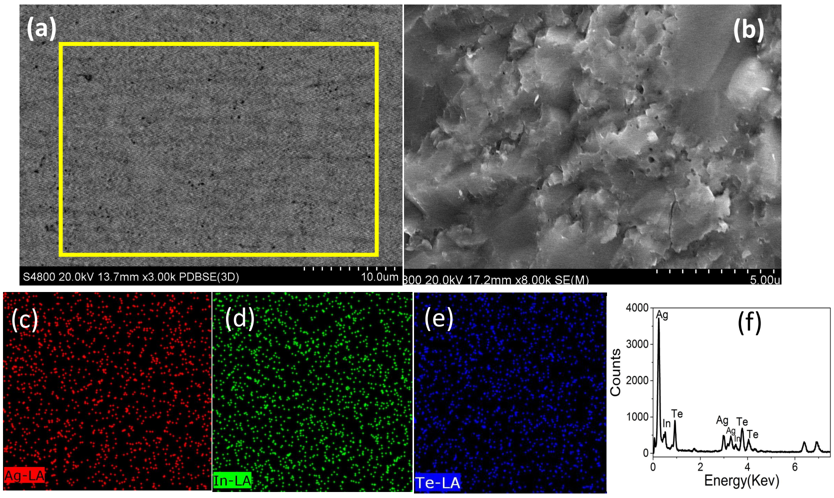

Figure S2 (a) SEM image of polished surface of the sample $Ag_{1-x}InTe_2$ with x = 0.15; (b) SEM image of the freshly fractured surface of the sample; (c-e) EDS mappings of three elements, Ag, In and Te; (f) An EDS pattern.

Table S1 Analyzed average chemical compositions (relative molars) identified for $Ag_{1-x}InTe_2$ with x = 0.15 (taken from three mappings), where the number of Te moles is normalized to be 2.0.

Compound	Ag	In	Te
$Ag_{1-x}InTe_2$ (x=0.15)	0.87	1.06	2.0

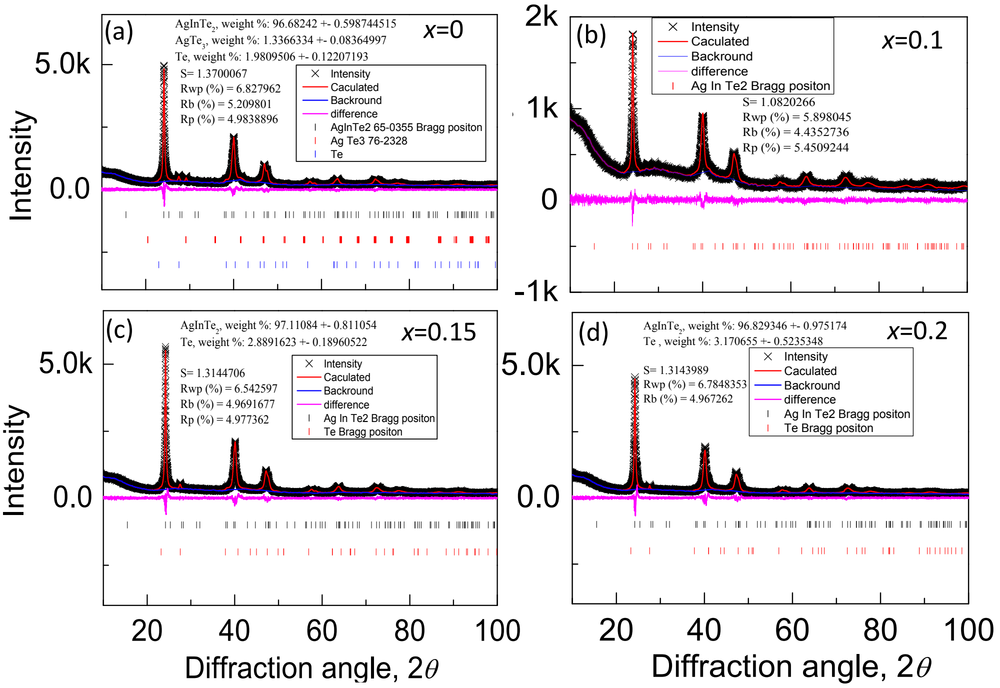

Figure S3 Rietveld refinement using the X-ray diffraction data for the samples $Ag_{1-x}InTe_2$ (x=0, 0.1, 0.15, 0.2).

Table S2 Experimental parameters of powder X-ray diffraction, and refined crystallographic data for different *x* values in $AgInTe_2$ without heat treatment.

Parameters	*x*=0	*x*=0.05	*x*=0.10	*x*=0.15	*x*=0.20	*x*=0.15 (heat treatment)
a (Å)	6.4253(5)	6.4233(4)	6.4153(5)	6.4029(6)	6.3943(8)	6.394(2)
b (Å)	6.4253(5)	6.4233(4)	6.4153(5)	6.4029(6)	6.3943(8)	6.394(2)
c (Å)	12.626(1)	12.6230(1)	12.6140(1)	12.6120(2)	12.601(2)	12.614(7)
V (Å^3)	521.26(6)	520.81(5)	519.14(6)	517.05(10)	515.22(15)	515.70(43)
η (=$c/(2a)$)	0.9825	0.9826	0.9831	0.9848	0.9850	0.9892
u	0.2588	0.2587	0.2585	0.25762	0.25739	0.2554
$d_{\mathrm{Ag\text{-}Te}}$	2.7994	2.7984	2.7944	2.7872	2.7831	2.7707
$d_{\mathrm{In\text{-}Te}}$	2.7336	2.7329	2.7310	2.7305	2.7282	2.7308
R_B (%)	5.10	5.21	4.44	4.97	4.97	4.04
R_p (%)	5.17	4.98	5.45	4.98	5.16	4.83
R_{wp} (%)	6.58	6.83	5.90	6.54	6.78	5.16
S	1.27	1.37	1.08	1.31	1.31	1.07

Table S3 Wyckoff Positions, Atomic Coordinates, and Occupancies of $AgInTe_2$ without heat treatment.

x value	Atom	Site	x	y	z	Biso (Å^2)	Occupancy
	In1	4b	0	0	1/2	0.026(5)	1
0	Te1	8d	0.2447(6)	1/4	1/8	0.011(1)	1
	Ag1	4a	0	0	0	0.055(8)	1
	In1	4b	0	0	1/2	0.022(5)	1
0.05	Te1	8d	0.2428(6)	1/4	1/8	0.014(1)	1
	Ag1	4a	0	0	0	0.112(5)	1
	In1	4b	0	0	1/2	0.060(1)	1
0.1	Te1	8d	0.2421(2)	1/4	1/8	0.003(4)	1
	Ag1	4a	0	0	0	0.042(3)	1
	In1	4b	0	0	1/2	0.016(3)	1
0.15	Te1	8d	0.2446(3)	1/4	1/8	0.004(2)	1
	Ag1	4a	0	0	0	0.040(5)	1
	In1	4b	0	0	1/2	0.013(1)	1
0.2	Te1	8d	0.2421(6)	1/4	1/8	0.006(2)	1
	Ag1	4a	0	0	0	0.053(1)	1

Table S4 Wyckoff Positions, Atomic Coordinates, and Occupancies of Te.

	Atom	Site	x	y	z	Biso (Å^2)	Occupancy
x=0	Te1	3a	0	0	1/3	0.035(3)	1
x=0.05	Te1	3a	0.2691(2)	0	1/3	0.025(2)	1
x=0.1	-----	-----	------	----	-----	------	------
x=0.15	Te1	3a	0.2692(2)	0	1/3	0.016(3)	1
x=0.2	Te1	3a	0.2691(2)	0	1/3	0.011(1)	1

Table S5 Wyckoff Positions, Atomic Coordinates, and Occupancies of $AgTe_3$.

	Atom	Site	x	y	z	Biso (Å^2)	Occupancy
x=0	Ag1	3a	0	0	0	0.026(2)	1
	Te1	9b	0	0.8328(1)	0.3413(2)	0.037(4)	1
x=0.05	Ag1	3a	0	0	0	0.024(3)	1
	Te1	9b	0.1672(1)	0.8328(2)	0.3413(2)	0.036(4)	1
x=0.1	----	----	----	----	----	----	----
	----	----	----	----	----	----	----
x=0.15	----	----	----	----	----	----	----
	----	----	----	----	----	----	----
x=0.2	----	----	----	----	----	----	----
	----	----	----	----	----	----	----

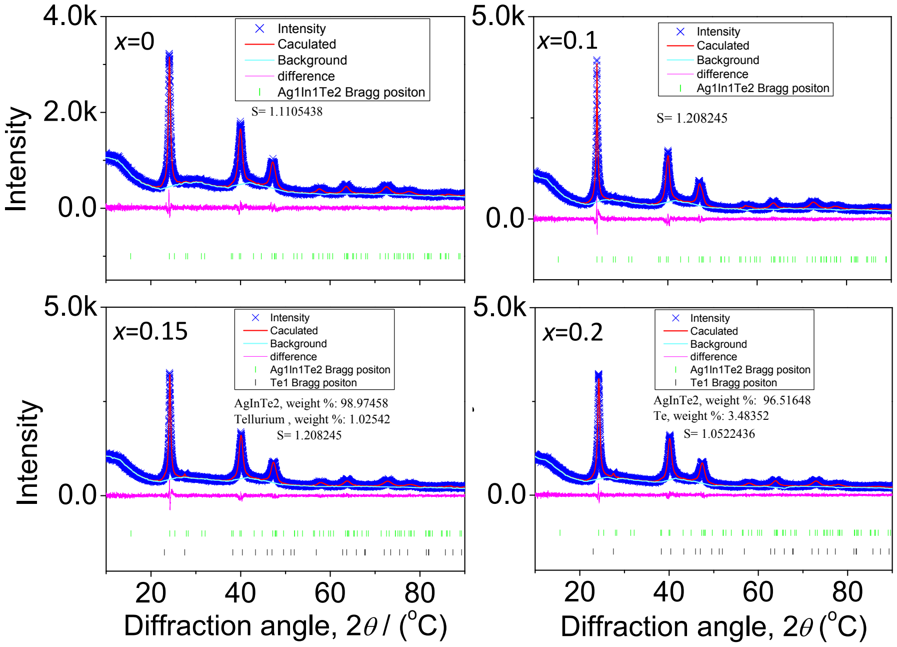

Figure S4 Rietveld refinement using the X-ray diffraction data for the samples $Ag_{1-x}InTe_2$ (x=0, 0.1, 0.15, 0.2) after annealing at 813 K for 72 h.

Table S6 Wyckoff Positions, Atomic Coordinates, and Occupancies of $AgInTe_2$ with heat treatment at 813 K for 72 h.

Parameters	x=0	x=0.10	x=0.15	x=0.20
a (Å)	6.409(8)	6.404(2)	6.394(2)	6.375(2)
b (Å)	6.409(8)	6.414(2)	6.394(2)	6.375(2)
c (Å)	12.638(5)	12.624(6)	12.614(7)	12.613(7)
V (Å^3)	519.11(32)	518.93(41)	515.70(43)	512.60(43)
η (=c/(2a))	0.9859	0.9856	0.9864	0.9892
u	0.2570	0.2572	0.2568	0.2554
$d_{Ag\text{-}Te}$	2.7888	2.7869	2.7818	2.7707
$d_{In\text{-}Te}$	2.7362	2.7331	2.7310	2.7307
R_B (%)	4.04	4.55	4.04	4.046
R_p (%)	4.56	4.87	4.83	4.83
R_{wp} (%)	5.07	5.88	5.16	5.16
S	1.11	1.21	1.07	1.07

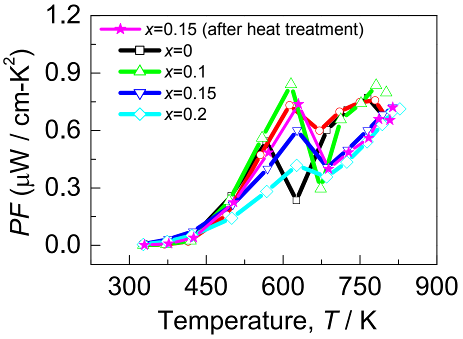

Figure S5 Power factors (PF) as a function of temperature for different x values in $Ag_{1-x}InTe_2$. The power factor of the sample at x=0.15 after heat treatment at 813 K is presented for comparison.
